# Microbiome on the Bone-Anchored Hearing System: A Prospective Study

**DOI:** 10.3389/fmicb.2019.00799

**Published:** 2019-04-26

**Authors:** Tim G. A. Calon, Margarita Trobos, Martin L. Johansson, Joost van Tongeren, Malieka van der Lugt-Degen, A. M. L. Janssen, Paul H. M. Savelkoul, Robert J. Stokroos, Andries E. Budding

**Affiliations:** ^1^Department of Otorhinolaryngology, Head and Neck Surgery, Maastricht University Medical Center, Maastricht, Netherlands; ^2^Department of Biomaterials, Institute of Clinical Sciences, Sahlgrenska Academy, University of Gothenburg, Gothenburg, Sweden; ^3^Oticon Medical AB, Askim, Sweden; ^4^IS-Diagnostics Ltd., Amsterdam, Netherlands; ^5^Department of Methodology and Statistics, Care and Public Health Research Institute, Maastricht University, Maastricht, Netherlands; ^6^Amsterdam UMC, Vrije Universiteit Amsterdam, Department of Medical Microbiology and Infection Control, Amsterdam, Netherlands; ^7^Department of Medical Microbiology, NUTRIM School of Nutrition and Translational Research in Metabolism, Maastricht University Medical Center, Maastricht, Netherlands; ^8^Department of Otorhinolaryngology, Head and Neck Surgery, University Medical Center Utrecht, Utrecht, Netherlands; ^9^Brain Center Rudolf Magnus, University Medical Center Utrecht, Utrecht, Netherlands

**Keywords:** bone-anchored devices, percutaneous implant, BAHS, microbiome, Holgers Index, IS-pro

## Abstract

The bone-anchored hearing system (BAHS) has evolved to a common treatment option for various types of hearing revalidation. The BAHS consists of an implant in the skull that breeches the skin. Soft tissue reactions are a common complication associated with BAHS and are generally poorly understood. This study aims to investigate the influence of BAHS and associated skin reactions around the implant. A total of 45 patients were prospectively followed from implantation up to at least 1 year. Swabs were obtained at baseline, 12 weeks follow-up and during cases of inflammation (Holgers score ≥2). The microbiota was assessed using IS-pro^TM^, a bacterial profiling method based on the interspace region between the 16S–23S rRNA genes. Detection of operational taxonomic units, the Shannon Diversity Index, sample similarity analyses and Partial Least Squares Discriminant Analysis (PLS-DA) were employed. *Staphylococcus epidermidis*, *Streptococcus pneumoniae/mitis*, *Propionibacterium acnes*, *Staphylococcus capitis*, *Staphylococcus hominis*, *Bifidobacterium longum*, *Haemophilus parainfluenzae*, *Lactobacillus rhamnosus*, *Bordetella* spp., *Streptococcus sanguinis*, *Peptostreptococcus anaerobius*, *Staphylococcus aureus*, *Lactococcus lactis*, *Enterobacter cloacae*, and *Citrobacter koseri* were the most commonly found bacterial species. *S. pneumoniae/mitis* was significantly more often observed after implantation, whereas *P. acnes* was significantly less observed after implantation compared with baseline. The relative abundance of *S. epidermidis* (17%) and *S. aureus* (19.4%) was the highest for the group of patients with inflammation. The Shannon Diversity Index was significantly increased after implantation compared with pre-surgical swabs for Firmicutes, Actinobacteria, Fusobacteria, Verrucomicrobia (FAFV), but not for other phyla. When combining all phyla, there was no significant increase in the Shannon Diversity Index. The diversity index was similar post-surgically for patients experiencing inflammation and for patients without inflammation. With a supervised classifier (PLS-DA), patients prone to inflammation could be identified at baseline with an accuracy of 91.7%. In addition, PLS-DA could classify post-surgical abutments as non-inflamed or inflamed with an accuracy of 97.7%. This study shows the potential of using IS-pro^TM^ to describe and quantify the microbiota associated with the percutaneous BAHS. Furthermore, the results indicate the possibility of an early identification of patients susceptible to adverse skin reaction following implantation. Both *S. aureus* and *S. epidermidis* should be considered as relevant bacteria for BAHS-associated inflammation.

## Introduction

The bone-anchored hearing system (BAHS) was introduced in 1977 (Tjellström et al., 1981). The BAHS is a retro-auricular titanium implant that is fixed in the skull via osseointegration. A skin-penetrating abutment is placed on the implant allowing the coupling of a sound processor (Tjellström et al., 1981) (Supplementary Figure S1). The BAHS is considered a successful treatment option with overall good outcomes. It is an established therapy for patients suffering from several types of hearing loss including conductive hearing loss, mixed hearing loss, and single sided deafness (Snik et al., 2005; Faber et al., 2015). During the last years, treatment options have become less invasive, resulting in improved outcomes regarding esthetics, pain, numbness of the skin, implant survival, and soft tissue reactions (Hultcrantz, 2013; den Besten et al., 2016; Calon et al., 2018). Soft tissue reactions, such as inflammation of the peri-abutment skin, are still a common complication, but the majority of cases are relatively easily treated (Dun et al., 2012; Verheij et al., 2016).

Soft tissue reactions can reduce overall patient satisfaction, use of the sound processor and possibly quality of life. Additionally, patients require extra consultations in the out-patient clinic. Implant-associated infections represent a challenge for BAHS as well as for other medical devices (Busscher et al., 2012). The peri-abutment skin surrounding the BAHS is usually graded according to the Holgers Index, consisting of a five-point grading scale (Holgers et al., 1988). Several factors have been postulated to influence the etiology of adverse soft tissue reactions and inflammation, including implant design, strains and stresses in the peri-abutment tissue, surgical technique, immune responses, patient related factors, and biofilm formation (Monksfield et al., 2011; Johansson, 2018; Trobos et al., 2018). Most likely, a complex interplay between these factors exists.

The skin next to the abutment does not attach to the abutment, instead there is an epithelial down-growth with non-keratinized tissue (Holgers et al., 1995a). Post-BAHS surgery, the anatomical situation is different from that of normal skin due to the permanent breach of the skin. This specific skin-abutment transition zone could constitute a special niche containing a distinct bacterial microbiota that differs from the normal skin (Trobos et al., 2018).

In relation to BAHS, several different species of bacteria have been detected, and bacterial colonization of implants and abutments have been shown (Holgers et al., 1995b; Monksfield et al., 2011; Trobos et al., 2018). *Staphylococcus aureus* and coagulase-negative staphylococci (CoNS), *Bacteroides ureolyticus*, *Proteus*, *Klebsiella*, *Escherichia coli*, and *Peptostreptococcus* have been cultured from non-inflamed and inflamed BAHS (Holgers and Ljungh, 1999). *S. aureus* and *Staphylococcus epidermidis* can infect cells adjacent to the implant surface (Boelens et al., 2000; Broekhuizen et al., 2008b; Garzoni and Kelley, 2009). Moreover, peri-implant bacteria may even re-colonize an implant after antibiotic treatment, becoming a source of infection (Broekhuizen et al., 2008a).

Biofilms have been found on both non-inflamed and inflamed BAHS (Monksfield et al., 2011; van Hoof et al., 2015). Biofilms consist of layers of bacterial cells and their secreted extracellular polymeric substances, and are particularly resistant to antibiotics (Busscher et al., 2012). Recently, Buskermolen et al. (2018) showed that oral biofilms could be classified as either supporting oral wound healing or related to pathogenic responses. It is conceivable that similar mechanisms could play a role in soft tissue reactions in BAHS.

Bacteria, whether as single, part of biofilm or intra-cellular, are all sources of potential infection related to implants. Insight into the microbiota profiles and changes related to BAHS implantation and skin inflammation could increase the knowledge and understanding of the role of the microbiota in BAHS. This knowledge could be used to prevent skin inflammation and improve treatment directed at specific pathogens.

In this study, we evaluated the microbiota profiles and their changes by using IS-pro^TM^, a molecular technique based on profiling of the bacterial 16S–23S ribosomal interspace region (Budding et al., 2010). This study aims to (I) evaluate the bacterial changes induced by BAHS surgery and (II) to identify a microbiota profile associated with peri-abutment skin inflammation.

## Materials and Methods

### Ethics Statement

This study is part of a larger trial comparing two surgical techniques for installing BAHS (Calon et al., 2016) and the short-term clinical results have recently been published (Calon et al., 2018). This study was performed in accordance with the Dutch legislation of Medical Research Involving Human Patients Act and with the ethical standards on human experimentation in the Netherlands. The study was conducted in accordance with the Declaration of Helsinki and was approved by the medical ethical committee of Maastricht University Medical Centre + (MUMC+) (NL50072.068.14) and registered at clinicaltrials.gov (NCT02438618). Consent procedure was in accordance with the study protocol and ISO 14155.

### Population

Patients were recruited at the out-patient department of otorhinolaryngology of MUMC+. All patients were enrolled in a randomized controlled clinical trial comparing two surgical BAHS techniques. All subjects were scheduled to receive a Ponto Wide implant with a pre-mounted abutment (Oticon Medical AB, Askim, Sweden) using single stage surgery (Westerkull, 2011; Calon et al., 2016). Patients had to be older than 18 years and qualify to undergo unilateral BAHS surgery. Exclusion criteria were: (I) a history of immunosuppressive disease, (II) usage of systemic immunosuppressive medication, (III) bilateral BAHS placement, (IV) relevant dermatological disease such as psoriasis or severe eczema, (V) participation in other studies, and (VI) when no suitable site for a 4-mm Ponto Wide implant was found during surgery. All patients provided written informed consent. Complete clinical data, including additional patients not participating in the swab collection, have been published previously (Calon et al., 2018). This paper reports on the subset of patients where swab samples were collected.

### Procedures

Baseline characteristics including sex, age, body mass index, smoking habits, medical history, and medication were recorded in the case report forms. These case report forms were designed to record clinical information on patients for this specific study (Calon et al., 2016). Both the linear incision technique with soft tissue preservation (Hultcrantz, 2011; Calon et al., 2016) and the Minimally Invasive Ponto Surgery (MIPS) technique were used for BAHS implantation surgery (Calon et al., 2016; Johansson et al., 2017). For 9 days following surgery, a healing cap and gauze drenched in antibiotic ointment (Terra-Cortril, Pfizer, New York, NY, United States) were applied on the abutment.

Patients attended standard follow-up visits at 9 days, 3 weeks, 12 weeks, and 1 year. Prior to surgery, samples for bacterial analyses were obtained from (i) the intended implantation site (baseline implant skin sample, BIS) and (ii) contra-lateral control side (baseline control sample, BCS). Before BIS was taken, the hair was shaved. No antiseptics were used. A swab was used to obtain a sample from an area of 2 by 2 cm at the intended implant site. The BCS was obtained at the retro-auricular area without any prior cleaning or shaving. A swab was used to obtain a sample from an area of 2 by 2 cm. After the BIS was obtained, the skin was cleaned with antiseptics before surgery was commenced. At 9-days, patients received written and oral instruction regarding the maintenance of the implant. They were instructed to gently clean the implant each day using plain water and a toothbrush provided by the manufacturer.

During follow-up, swab samples were obtained from the peri-abutment skin site (peri-abutment sample, PAS) and contra-lateral control site (contra-lateral sample, CLS) at (i) 12 weeks and (ii) during episodes of inflammation. The PAS was obtained by swabbing the external side of the abutment 360°. Thereafter, the same swab was immediately used to swab approximately 1 cm of peri-abutment skin 360° around the abutment. If peri-abutment fluid (e.g., moist) was present at the peri-abutment skin, this was obtained as well. No cleaning was performed before the sample was obtained. The CLS was obtained in a similar manner as the BCS where 2 by 2 cm of unshaved skin was swabbed at the contralateral side of the abutment without prior cleaning or antiseptics.

For BAHS, the Holgers Index is used to assess the peri-abutment skin. It is a five-grade scale where; 0 No irritation; 1 Slight redness; 2 Red and slightly moist tissue, no granuloma formation; 3 Reddish and moist; sometimes granulation tissue; and 4 Removal of skin-penetrating implant necessary due to infection (Holgers et al., 1988). In the context of this study, peri-abutment skin inflammation was considered present if the patient was rated as having a Holgers Index score of two or higher. If the patient demonstrated peri-abutment skin-inflammation (Holgers Index score ≥2), either at a standard follow-up visit or an extra consultation, extra swabs were taken at the inflamed peri-abutment site (iPAS) and the contra-lateral site (CLS). Samples were stored in an Eppendorf container with 200 μL Transportbuffer (IS-Diagnostics, Amsterdam) at -20°C.

For DNA extraction an easyMAG machine (bioMérieux Clinical Diagnostics, Marcy-l’Etoile, France), an automated system for total nucleic acid isolation, was used. To every sample, 500 μL of nucliSENS lysis buffer was added. This suspension was vortexed for 5 min at ≥1400 rpm and subsequently centrifuged at 18000 *g* for 2 min. The complete volume was transferred to an 8-welled easyMAG container and 2 mL nucliSENS lysis buffer was added. After incubation at room temperature for ≥10 min, 70 μL of magnetic silica beads were added. Afterwards, the mixture was inserted in the easyMAG machine and the “specific A” protocol was chosen, selecting the off-board workflow and eluting DNA in 70 μL of buffer. All extracted DNA was stored at 4°C.

### IS-pro^TM^ Profiling of Microbiota

Isolated DNA was further processed as recommended by the manufacturer according to the IS-pro^TM^ assay (IS-Diagnostics, Amsterdam, Netherlands). Briefly, the IS-pro^TM^ technology is a bacterial profiling technique, which is based on detection and categorization of the species-specific length differences of the 16S–23S rRNA gene interspacer region of bacteria. This region is located at the end of 16S and beginning of 23S varying between 200 and 2000 base pairs in length. The differences in length are detected after amplification of the IS region. After eubacterial amplification of the IS region the subsequent amplicons are separated on a capillary sequencer and subsequently compared to the database including the combination of amplicon length and species. The technique has been standardized for clinical use in several other sample types which are previously published (Budding et al., 2010; Rutten et al., 2015; de Meij et al., 2016; Cranendonk et al., 2018). Basically, the IS-pro technology detects all bacterial species at the DNA level in a clinical sample. The IS-pro technology has been validated against NGS 16S sequencing and culture showing very high correlations. In addition IS-pro is a rapid, easy to perform standardized microbiota profiling technology enabling discrimination at the phylum, genus and species level (Budding et al., 2016; de Meij et al., 2016). In practice the procedure consists of two standardized multiplex PCR amplifications. The first PCR is specific for Firmicutes, Actinobacteria, Fusobacteria, Verrucomicrobia (FAFV), and Bacteroidetes. FAFV includes many skin bacterial species. The second PCR is specific for Proteobacteria. Amplifications were carried out on a GeneAmp PCR system 9700 (Applied Biosystems, Foster City, CA, United States). Cycling conditions for PCR were: 10 cycles at 94°C for 30 s, 67°C (1°C decrease per cycle) for 45 s, and 72°C for 1 min; followed by 25 cycles at 94°C for 30 s, 57°C for 45 s, and 72°C for 1 min; and the final step at 72°C for 11 min and cooling down to 4°C. A total of 5 μL of PCR amplification product was mixed with 20 μL of IS-pro^TM^ eMix (IS-Diagnostics). PCR fragment separation was performed on an ABI Prism 3500 genetic analyzer (Applied Biosystems). The resulting peak profiles with the species specific peaks were uploaded to the database for species identification.

### Data Analysis and Statistics

Data were analyzed using the proprietary IS-pro^TM^ software (IS-Diagnostics, Amsterdam, Netherlands) and R version 3.3.2 (R Foundation for Statistical Computing, Vienna, Austria). Statistical significance was assumed at *p* ≤ 0.05. Data was visualized in Spotfire version 7.10 (TIBCO, Palo Alto, CA, United States). Species were identified according to IS-pro^TM^ fragment profiles, with the use of IS-pro^TM^ software. Heatmaps were created by hierarchical clustering of samples by the unweighted pair group method with arithmetic mean (UPGMA).

#### Prevalence of Species

Prevalence of the most common bacterial species on BIS and PAS at 12-weeks were compared using McNemar’s test. Samples obtained from non-inflamed sites (PAS) were compared with samples obtained from cases with inflammation (iPAS) using McNemar’s test (McNemar, 1947). This test compares proportions over time in paired samples. For patients with several episodes of inflammation, only the first swab obtained during the first episode of inflammation was used in the analysis.

#### Sample Similarity

Log2 transformed microbiota profiles were compared pairwise within and between patients with cosine correlation coefficients for all bacteria and FAFV (Daniels et al., 2014; de Meij et al., 2016). Mean (*M*) and Standard Deviation (*SD*) were calculated.

#### Sample Diversity

To determine bacterial diversity within patients, the Shannon Diversity Index results were computed for all bacteria. The Shannon Diversity Index changes over time within patients were evaluated using paired sample *t*-test. Between-subject comparisons (no inflammation vs. inflammation) were evaluated using an independent samples *t*-test. To correct for multiple testing, the Bonferroni method was used for the number of phyla tested. All distributions were checked for normality. In case of non-normality, the non-parametric Wilcoxon signed-rank test or Mann–Whitney *U*-test was performed.

#### Partial Least Square Discriminant Analysis

Partial least squares discriminant analysis (PLS-DA) was used to classify swabs for clinical status. PLS-DA entails an algorithm-based classification method designed to identify Operational Taxonomic Units (OTU) as predictors for predefined classifications (Perez-Enciso and Tenenhaus, 2003; Blaxter et al., 2005; Rajilic-Stojanovic et al., 2011; Daniels et al., 2014). Classifications included inflammation status for PAS (PAS vs. iPAS) and future inflammation during follow-up for BIS. The Variable Importance for Projection (VIP) criterion was used to identify OTUs that discriminate between groups. A VIP score >1.2 was considered as the relevant threshold (Perez-Enciso and Tenenhaus, 2003; Modlich et al., 2005; Daniels et al., 2014).

## Results

### Patient Characteristics

From December 2014 to January 2017, 49 patients were included. Four patients were excluded due to implant loss during follow-up. For one patient, the baseline swab was missing, and this patient was therefore excluded from analyses involving baseline implantation site swabs. Patient characteristics are summarized in Table 1. Mean age was 52 years (*SD* = 15). Mean Body Mass Index was 28 kg/m^2^ (*SD* = 6). Ten patients were smokers (22%) and 35 were non-smokers (78%). A total of 22 (49%) patients received a BAHS using the MIPS procedure and 23 (51%) patients received a BAHS using the linear incision technique with soft tissue preservation. During the study, 12 (27%) patients experienced at least one episode of inflammation.

**Table 1:** Patient characteristics.

Characteristics	(*n* = 45)
Age (years)	52 (15)
Gender	
Male	18 (40%)
Female	27 (60%)
Body mass index	28 (6)
Diabetes	6 (13%)
Smoking	
Non-smoking	35 (78%)
Smoking	10 (22%)
Surgical technique	
MIPS	22 (49%)
Linear incision with soft tissue preservation	23 (51%)
At least one episode of inflammation	12 (27%)
At least two episodes of inflammation	5 (11%)


*Mean (*SD*) is presented for continuous variables. Number (%) is presented for categorical variables.*

### Species

**FIGURE 1:**
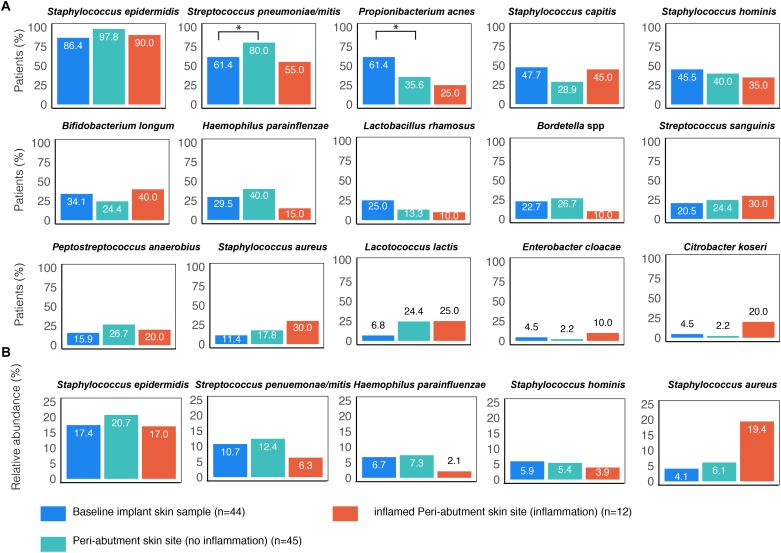
Prevalence of the most commonly found bacterial species associated with BAHS. Sample sites: Baseline implant skin (BIS), non-inflamed peri-abutment skin (PAS, Holgers Index score <2) and inflamed peri-abutment skin (iPAS, Holgers Index score ≥2). **(A)** The percentage of patients with observed bacterial species is presented. **(B)** Relative abundance (in percentage) of five commonly found species based on the total bacterial counts detected in the three sample types: baseline implant skin sample (BIS), peri-abutment skin site (PAS), and inflamed PAS (iPAS). ^∗^Indicates *p*-value ≤0.05.

An overview of the ten most commonly found bacteria on the BIS, PAS (Holgers score ≤1) and iPAS (inflamed peri-abutment skin/Holgers Index score ≥2) is presented in Figure 1A. *S. epidermidis* (found in 86.4–97.8% of patients) followed by *Streptococcus pneumoniae/mitis* (found in 55–80% of patients) were the two most commonly observed species for all three types of samples. *Propionibacterium acnes* (61.4%) and the two CoNS species *Staphylococcus capitis* (47.7%) and *Staphylococcus hominis* (45.5%) were commonly observed on the BIS. *S. hominis* (40%), *Haemophilus parainfluenzae* (40%), and *P. acnes* (35.6%) were commonly found on non-inflamed PAS. During inflammation, the peri-abutment site (iPAS) was frequently colonized by *S. capitis* (45%), *Bifidobacterium longum* (40%), *S. hominis* (35%), *S. aureus* (30%), and *Streptococcus sanguinis* (30%). McNemar’s test revealed that the PAS had significantly more *S. pneumoniae/mitis* (*p* = 0.03) and significantly less *P. acnes* (*p* = 0.02) compared with the BIS. McNemar’s test between PAS and iPAS demonstrated no significant differences for the amount of *S. aureus* (*p* = 0.62), *P. acnes* (*p* = 1.0) or any of the other bacterial species within-subjects.

The relative abundances of *S. aureus* were 19.4% for the patients with inflammation (iPAS) compared with 6.1% for the patients without inflammation PAS (Figure 1B). However, *post hoc*, Wilcoxon signed-rank tests revealed no significant differences in relative abundances for *S. aureus* (*p* = 0.21) or any of the other species when comparing non-inflamed PAS with inflamed PAS.

The relative abundances of *S. epidermidis* were 17% for patients with inflammation (iPAS) compared with 20.7% for patients without inflammation PAS. Other bacteria such as *S. hominis* and *S. pneumoniae/mitis* showed a lower contribution to the complete bacterial load observed on the abutment during cases of inflammation.

### Diversity Analysis

**FIGURE 2:**
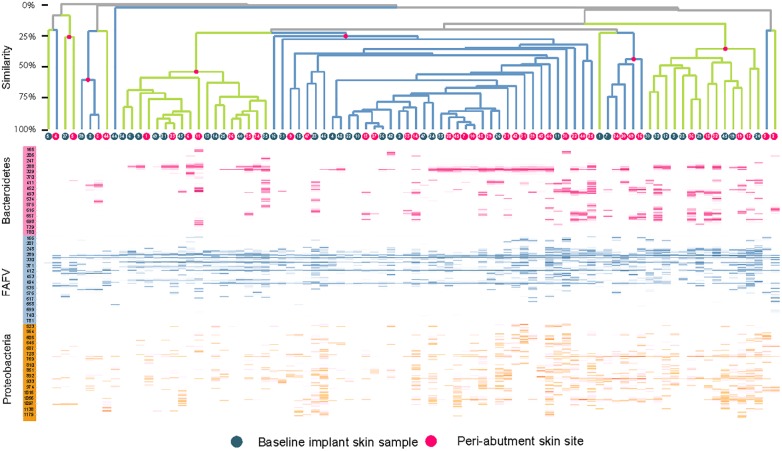
Clustered heatmap with IS-pro profiles for baseline implant skin samples and peri-abutment skin sites obtained at 12-week follow-up. Red dots and branches indicate the percentage of similarity between swabs below. Swabs are clustered per phylum. FAFV represents the phyla Firmicutes, Actinobacteria, Fusobacteria, and Verrucomicrobia. Patient identification numbers are indicated in dots. All baseline implant skin samples and all 12-week peri-abutment skin site swabs are included. Overall no specific pattern can be observed distinguishing baseline implant skin samples and peri-abutment skin sites or patient clusters.

**FIGURE 3:**
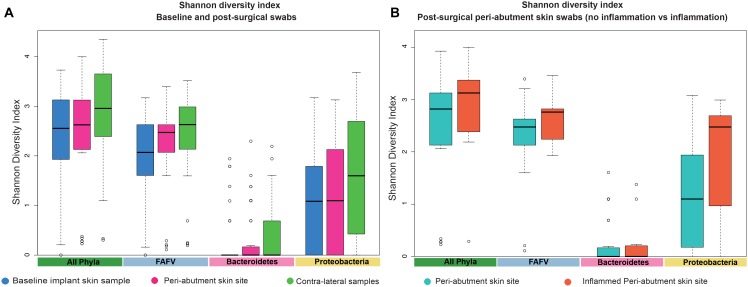
Shannon Diversity Index. **(A)** The Shannon Diversity Index for baseline implant skin samples, 12-week peri-abutment skin site swabs, and contra-lateral samples obtained at 12-week follow-up. **(B)** The Shannon Diversity Index for non-inflamed peri-abutment skin swabs at 12 weeks and inflamed peri-abutment skin swabs obtained during follow-up.

The UPGMA clustered heat map comparing BIS to PAS is presented in Figure 2. This analysis was performed at the phylum level to obtain a more general overview on specific microbiota shifts at this level in addition to the more specific species level. No clear distinction between 12-week PAS and BIS could be observed. Although one 12-week PAS cluster was observed, BIS and 12-week PAS for the same patient generally did not cluster together. The Shannon Diversity Index for the BIS, PAS, iPAS, and CLS is presented in Figure 3. For all bacteria combined, there was no significant difference in the Shannon Diversity Index between PAS at 12 weeks and BIS (*p* = 0.16). However, the PAS at 12-week follow-up showed a significantly higher diversity index for bacteria from the FAFV group compared with the BIS (*p* = 0.04). Within-subject analyses showed no significant differences for Proteobacteria and Bacteroidetes between BIS and PAS at 12-weeks. The comparative analyses between PAS and iPAS showed no significant differences for FAFV, Proteobacteria or Bacteroidetes.

### Sample Similarity

All similarity results are presented in Table 2. At baseline, the similarity within patients was 59% for all bacteria combined and 77% for FAFV, and between patients the similarity was lower with 43 and 60%, respectively. Baseline implant skin and peri-abutment skin samples showed higher similarities within patients for all bacteria combined (45%) and FAFV (62%) compared to between-subject similarities for all bacteria (38%) and FAFV (55%). Small differences were observed between PAS and iPAS within subject similarities.

**Table 2:** Sample similarities.

Percentage of sample similarities	All bacteria (SD)	FAFV (SD)
**Baseline**		
**Similarity between baseline implant skin sample (BIS) and contra-lateral samples (*n* = 10)**		
Intra-subject	59 (21)	77 (14)
Inter-subject	43 (15)	60 (12)
**12-week follow-up**		
**Similarity between peri-abutment skin site (PAS) and baseline implant skin sample (BIS) (*n* = 44)**		
Intra-subject	45 (13)	62 (16)
Inter-subject	38 (11)	55 (13)
**Peri-abutment skin site (intra-subject)**		
**Similarity between peri-abutment skin site (PAS, iPAS) and baseline implant skin sample (BIS)**		
No inflammation (*n* = 32)	46 (0.13)	63 (15)
Inflammation (*n* = 12)	44 (0.19)	62 (15)
**Similarity between peri-abutment skin site (PAS, iPAS) and contra-lateral samples (CLS)**		
No inflammation (*n* = 32)	48 (0.13)	68 (13)
Inflammation (*n* = 12)	57 (19)	70 (19)


*Similarity coefficients (standard deviations) obtained from Log2 data are presented.*

#### Partial Least Square Discriminant Analysis

**FIGURE 4:**
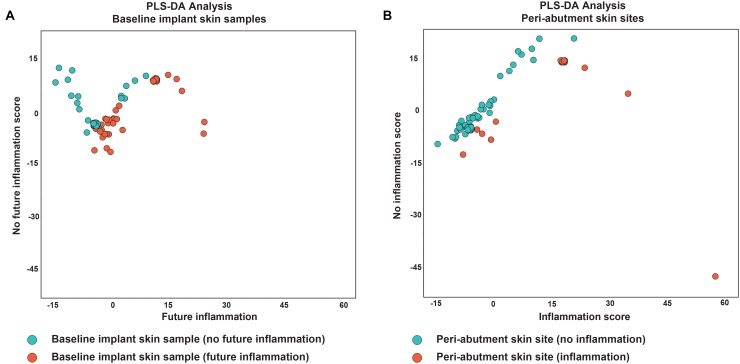
Partial least square discriminant analysis. **(A)** Analysis of baseline implant skin samples with no inflammation (green) during the 1-year follow-up. Red dots indicate baseline implant skin samples (BIS) with inflammation during X months follow-up. **(B)** Analysis of peri-abutment skin site swabs (PAS) obtained at 12-week follow-up with no inflammation during X months follow-up (green dots) and peri-abutment skin site swabs obtained during inflammation (iPAS) (red dots). Inflammation was defined as a Holgers Index score ≥2. On the *x*-axis the first component (future) inflammation, in the PLS-DA model is displayed. On the *y*-axis the second component, no (future) inflammation, is displayed.

Results for the PLS-DA analyses are presented in Figure 4. The first PLS-DA analysis aimed to predict patients prone to inflammation. For all the 32 patients not experiencing inflammation during the follow-up period, all 32 BIS swabs were classified as no inflammation in the future. For the 12 patients experiencing inflammation, 9 of the 12 BIS swabs were correctly classified in the group experiencing inflammation in the future. Nine out of 12 baseline swabs were correctly classified in the group experiencing inflammation in the future. This yielded a sensitivity of 75%, specificity of 100% and accuracy of 93.3% for BIS classified as prone to inflammation in the future. The second PLS-DA analysis aimed to identify iPAS. Thirty-three cases of non-inflamed PAS and 11 of 12 cases of iPAS were correctly classified as non-inflamed and inflamed, respectively, resulting in a sensitivity of 91.7%, specificity of 100%, and accuracy of 97.7%.

## Discussion

### Species

In this study, BAHS recipients were followed for at least 1 year. To date, the influence of implantation of the skin penetrating BAHS on the skin microbiota has remained elusive. By employing a validated molecular based profiling technique, we could provide an overview of the microbiota for the skin of the retro-auricular crease and the peri-abutment skin, both under normal conditions and in cases of inflammation.

Previous work has mainly evaluated the microbiology of BAHS using conventional culture techniques (Holgers and Ljungh, 1999; Trobos et al., 2018). In line with our results, Holgers scorings of 0 and 1 have been shown to be mainly associated with coagulase-negative staphylococci (CoNS), *P. acnes* and *S. aureus* (Holgers and Ljungh, 1999). CoNS include common skin bacteria such as *S. epidermidis*, *S. capitis*, and *S. hominis*. During healthy and inflammatory conditions, CoNS (specially *S. epidermidis*) and *S. aureus* have been cultured (Holgers et al., 1992; Holgers and Ljungh, 1999; Trobos et al., 2018) and their presence was further confirmed in this study using a molecular method. Therefore, *S. aureus* and *S. epidermidis* should be considered as relevant bacteria associated with BAHS. In fact, *S. epidermidis*, as part of the native flora of the skin, may be introduced to the site when the skin is breached by the abutment, explaining its increased presence at the site where the abutment penetrates the skin. Furthermore, the ability of *S. epidermidis* to form biofilms enables it to adhere to medical devices and be protected from the patient’s immune response and antibiotics (Mack et al., 1996). This could also have contributed to the increased presence of *S. epidermidis* on and around the abutment compared with baseline.

During cases of peri-abutment inflammation, the numbers of *S. aureus*, *S. epidermidis*, and *S. pneumoniae/mitis* that were isolated were increased, with *S. aureus* as the most abundant bacterium. Especially compared with baseline and non-inflamed PAS, the relative presence of *S. aureus* on iPAS was striking. Interestingly, we observed a decrease in *S. epidermidis* and an increase of *S. aureus* during inflammation. Using PLS-DA analysis, it was possible to classify patients prone to inflammation and inflamed abutments with a high accuracy. Therefore, the IS-pro^TM^ technique could have clinical usefulness in the early detection of susceptible patients to inflammation and serve as a tool for the follow-up of patients at risk for soft tissue complications.

### Correlations and Diversity

At baseline, the similarity analysis between implant site swabs and skin controls for all bacteria was higher within patients than between patients, meaning that within each subject the microbiota in both retro-auricular creases was similar. Furthermore, at baseline a strong similarity was found within patients for FAFV. This indicates that within patients the Gram-positive fraction of the microbiota on both sides of the retro-auricular head strongly correlates. Based on descriptive statistics, the correlation results indicate that the microbiota from samples obtained over time is more similar within patients than between patients in this study group. This observation has been previously reported using 16S rRNA metagenomic sequencing showing less intrapersonal variation in microbiota between symmetrical skin sites than the interpersonal variation (Gao et al., 2007; Costello et al., 2009; Grice et al., 2009). It has been suggested that external environmental factors (climate and geography), host factors (immune status and pathophysiology) and historical exposures may account for the interpersonal variation (Grice and Segre, 2011).

In addition, within patients, moderate to strong correlations of FAFV between the peri-abutment skin and the baseline implant skin as well as the baseline control sample was demonstrated in this study. In contrast, a weaker correlation was observed between patients. Differences in cosine correlations between non-inflamed PAS and iPAS were small with only 1–2%. Our results could indicate that within-subjects the microbiota is relatively stable. Within the limitations of our study, both a similar normal skin microbiota and a slightly less similar abutment microbiota seem to exist between individuals. Moreover, the Shannon Diversity index significantly increased for skin bacteria after BAHS implantation. These results are in line with previous observations, indicating that diversity of skin bacteria increases after BAHS implantation (Trobos et al., 2018).

### Peri-Abutment Skin Reactions

In clinical practice, patients after receiving a BAHS visit the hospital only once or twice after implantation and for a 5-year check-up. Whether annual check-ups are carried out or not varies per hospital. Adverse peri-abutment soft tissue reactions are reasons for extra visits to the hospital. Overall, patients that experience only one episode of inflammation can easily be treated with counseling regarding hygiene and local antibiotic treatment. However, patients with recurring soft tissue complaints are clinically challenging and associated with higher costs and morbidity (Dun et al., 2012; Verheij et al., 2016). Treatment of these cases typically involves prescription of oral antibiotics but may also require temporary abutment removal or extensive tissue revision surgery. If these individuals could be identified prior to implantation surgery, preventive measures may be employed or alternative rehabilitation treatments without a skin penetrating abutment could be considered, including softband, patch, and transcutaneous solutions. In this study only two patients experienced recurrent episodes of peri-abutment adverse skin reactions. This small number of patients limits the possibility to identify a specific microbiota for a larger clinically relevant population. In our practice the broad-spectrum antibiotic ointment Terra-Cortril, containing hydrocortisone, oxytetracycline, and polymyxin-B, is often prescribed for soft tissue reactions around BAHS. Due to its limited effect on staphylococci, in case of inflammation, it could be advisable to subscribe different ointments such as Mupirocin (Bactroban, GlaxoSmithKline, Brentford, London, United Kingdom) or Fucidin ointment that target *S. aureus* and *S. epidermidis* (EUCAST, 2018). When considering therapies targeted toward skin infection, they might not only require the inhibition of pathogenic bacteria, but at the same time to promote the growth of symbiotic bacteria (Grice et al., 2009). Alternative approaches such as probiotics could be considered for future research (Argenta et al., 2016).

### Sample Location

Recently, Trobos et al. (2018) demonstrated colonization of bacteria on the abutment, in the surrounding skin and in the peri-abutment fluid space on BAHS implanted in the skull. The microbiological profiles in the soft-tissue close to the abutment, in the space between the abutment and tissue and on the abutment over time yielded different total counts of selected bacterial groups. Results from the surrounding skin and peri-abutment space correlated most strongly with clinical outcome (Trobos et al., 2018). The microbiota profiles obtained in the current study may have been different if other sample types than swabs would have been included or if sampling would have been taken at specific compartments. With the technique used here, the swab touched the exterior part of the abutment, the skin surrounding the abutment and possibly the peri-abutment fluid.

### Strengths and Limitations of the Study

This is the first study investigating the effect of implantation and inflammation on the microbiota in association with BAHS using a molecular approach. The sample size is large considering this research field. The study design allowed the possibility to create a prediction model for patients prone to inflammation. For bacterial profiling, we employed IS-pro^TM^ which is a validated 16S–23S Interspacer PCR-based profiling method. IS-pro^TM^ uses 16S–23S region length in combination with specific polymorphisms to identify species per phylum. Phyla include FAFV, Bacteroidetes and Proteobacteria. This method allows for a fast analysis (<5 h) of bacteria from different human samples (Budding, 2016).

Unidentified bacteria were observed in this study of which the clinical significance has yet to be determined. The classification database can allow for some species to be unidentified since not all species are yet present in the database. The species list in the database is continuously updated by sequencing the unknown species. Markers for antibiotic sensitivity are not obtained using IS-pro^TM^. Therefore, isolation of the causative pathogen and its susceptibility testing would still be needed to guide targeted treatment. The skin microbiota not only consists out of bacterial species, but fungi might be important as well (Kong and Morris, 2017). The technique used in this study does not evaluate fungi.

In this study, only one type of implant system was evaluated and the observed results may differ when evaluating a different implant system. The overall hygiene at the site of the BAHS was evaluated during each visit. However, the information on the individual patient’s daily cleaning regimen was not obtained.

The swabs obtained during episodes of inflammation were collected at different time points. Hence, time-dependent changes might have influenced the results as well. In this study, only 12 patients experienced at least one episode of inflammation, which might explain the non-significant differences observed in inflamed and non-inflamed peri-abutment swabs. The supervised PLS-DA algorithm was able to identify most cases of inflamed peri-abutment swabs and baseline implant skin swabs that experienced a future inflammation. Due to the relatively small sample size these results warrant caution and should be cross-validated in a larger dataset to test for robustness. The peri-abutment skin was assessed using the Holgers Index. However, its biological or inter- and intra-observer reliability has been questioned (Calon et al., 2016; Kruyt et al., 2017).

## Conclusion

This study provides evidence for a microbiota associated with a bone-anchored hearing implant system. Particularly, *S. aureus* and *S. epidermidis* should be considered as relevant bacteria after implantation of the system. Furthermore, the results indicate the possibility of an early identification of patients with an increased susceptibility to adverse skin reaction.

Better understanding of the skin microbiota will help to elucidate (i) the microbial interdependencies necessary to maintain healthy skin conditions around BAHS, (ii) how specific bacterial species are involved in BAHS-associated infection and inflammation and (iii) facilitate the development of new antimicrobial strategies.

## Ethics Statement

This study was performed in accordance with the Dutch legislation of Medical Research Involving Human Patients Act and with the ethical standards on human experimentation in the Netherlands. The study was conducted in accordance with the Declaration of Helsinki and was approved by the medical ethical committee of Maastricht University Medical Centre + (MUMC+) (NL50072.068.14) and registered at clinicaltrials.gov (NCT02438618). Consent procedure was in accordance with the study protocol and ISO 14155.

## Author Contributions

TC involved in the execution and analysis of the study. MJ and JvT involved in the design and execution of the study. ML-D involved in the execution of the study. AJ involved in the design and analysis of the study. PS involved in the design of the study. RS supervised the study. AB involved in the design, execution, and analysis of the study. TC, MT, and MJ wrote the primary manuscript. All authors reviewed and edited the manuscript.

## Conflict of Interest Statement

The authors declare that this study received funding from Oticon Medical AB, Sweden (C50/NCT02438618). The funder had the following involvement with the study: study design and analysis. MJ is an employee of Oticon Medical AB. PS is a co-owner, shareholder and scientific advisor of the university spin-off company IS-Diagnostics Ltd. (Amsterdam, Netherlands). AB is also a co-owner of the university spin-off company IS-Diagnostics Ltd. (Amsterdam, Netherlands). ML-D and AB are employed by IS-Diagnostics Ltd. (Amsterdam, Netherlands). The remaining authors declare that the research was conducted in the absence of any commercial or financial relationships that could be construed as a potential conflict of interest. The reviewer HN declared a shared affiliation, with no collaboration, with several of the authors, MT and MJ, to the handling Editor at the time of review.

## Supplementary Material

FIGURE S1 Overview of a bone-anchored hearing system (BAHS) comprising a titanium implant and abutment. The sound processor is attached to the abutments proximal end **(A)**. The implant is installed in the temporal bone behind the ear. Vibrations generated by the sound processor are transmitted directly through the skull bone to the cochlea as bone conduction sound **(B)**. Images reproduced by kind permission of Oticon Medical AB©.
