# Supplementary figures and images for: Microbiome on the Bone-Anchored Hearing System: A Prospective Study
Source: Front Microbiol. 2019 Apr 26;10:799. doi: 10.3389/fmicb.2019.00799 (PMC6498861; doi:10.3389/fmicb.2019.00799)

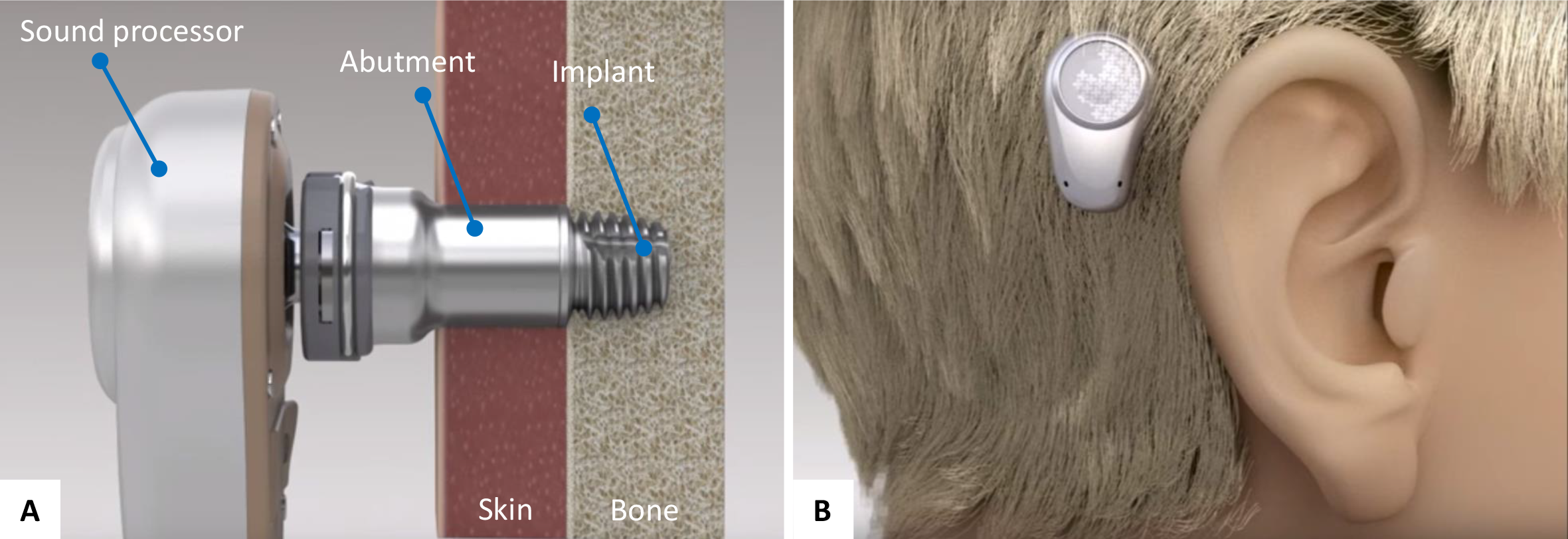

Supplement: FIGURE S1 — Overview of a bone-anchored hearing system (BAHS) comprising a titanium implant and abutment. The sound processor is attached to the abutments proximal end (A). The implant is installed in the temporal bone behind the ear. Vibrations generated by the sound processor are transmitted directly through the skull bone to the cochlea as bone conduction sound (B). Images reproduced by kind permission of Oticon Medical AB©. [file Image_1.TIF]
